# Novel AroA from *Pseudomonas putida* Confers Tobacco Plant with High Tolerance to Glyphosate

**DOI:** 10.1371/journal.pone.0019732

**Published:** 2011-05-18

**Authors:** Hai-Qin Yan, Su-Hua Chang, Zhe-Xian Tian, Le Zhang, Yi-Cheng Sun, Yan Li, Jing Wang, Yi-Ping Wang

**Affiliations:** 1 National Laboratory of Protein Engineering and Plant Genetic Engineering, College of Life Sciences, Peking University, Beijing, People's Republic of China; 2 Department of Histology and Embryology, Bengbu Medical College, Bengbu Anhui, People's Republic of China; 3 Key Laboratory of Mental Health, Institute of Psychology, Chinese Academy of Sciences, Beijing, People's Republic of China; 4 Institute of Pathogen Biology, Chinese Academy of Medical Sciences and Peking Union Medical College, Beijing, People's Republic of China; Cinvestav, Mexico

## Abstract

Glyphosate is a non-selective broad-spectrum herbicide that inhibits 5-enolpyruvylshikimate-3-phosphate synthase (EPSPS, also designated as AroA), a key enzyme in the aromatic amino acid biosynthesis pathway in microorganisms and plants. Previously, we reported that a novel AroA (PpAroA1) from *Pseudomonas putida* had high tolerance to glyphosate, with little homology to class I or class II glyphosate-tolerant AroA. In this study, the coding sequence of PpAroA1 was optimized for tobacco. For maturation of the enzyme in chloroplast, a chloroplast transit peptide coding sequence was fused in frame with the optimized *aroA* gene (*PparoA*1_optimized_) at the 5′ end. The *PparoA*1_optimized_ gene was introduced into the tobacco (*Nicotiana tabacum* L. cv. *W38*) genome via *Agrobacterium*-mediated transformation. The transformed explants were first screened in shoot induction medium containing kanamycin. Then glyphosate tolerance was assayed in putative transgenic plants and its T_1_ progeny. Our results show that the PpAroA1 from *Pseudomonas putida* can efficiently confer tobacco plants with high glyphosate tolerance. Transgenic tobacco overexpressing the *PparoA*1_optimized_ gene exhibit high tolerance to glyphosate, which suggest that the novel PpAroA1 is a new and good candidate applied in transgenic crops with glyphosate tolerance in future.

## Introduction

Currently, genetically modified (GM) crops are cultivated on the fields of 148 million hectares around the world [1]. Up to date, twenty-nine countries have approved planting of biotech crops and another thirty countries have approved import of biotech products for food and feed use. Globally, about 61% of GM crops are engineered for herbicide resistance (HR), including soybean, maize, canola, cotton, sugarbeet and alfalfa [1]. Glyphosate-resistant (GR) trait has been dominant in HR technology planted [2]. Since 1996, this trait has been rapidly adapted in soybean, cotton, maize and canola. GR crops marketed as Roundup Ready occupy the greatest acreage [2].

Glyphosate is a non-selective broad-spectrum herbicide that blocks plant growth by inhibiting 5-enolpyruvylshikimate-3-phosphate synthase (EPSPS, also designated as AroA) [3]. AroA is a key enzyme in the aromatic amino acid biosynthesis pathway in bacteria, fungi and higher plants [4]. Glyphosate also inhibits import of AroA into the chloroplast [5], which may contribute to the herbicide mode of action.

Transgenic plants engineered with glyphosate resistance have been developed by over-expression of wild-type AroA [6] or AroA enzymes with glyphosate resistance [7], [8], [9], [10]. Commercially, glyphsoate-resistant crops with acceptable levels of tolerance to the herbicide have been obtained only using the latter approach [10].

There are two classes of glyphosate-resistant AroA that have been reported. Class I AroA, originally a glyphosate-sensitive enzyme, can be converted into its glyphosate-tolerant form by introducing a point mutation. Glyphosate tolerance in transgenic tobacco was first reported by expressing the P101S substitution mutant of *Salmonella typhimurium* AroA [7], [11]. Another case was a G96A substitution mutant of AroA in transgenic petunia [12], [13]. There were other genetic manipulations of bacterial AroA to reduce its affinity to glyphosate and conferred transgenic plants for glyphosate-tolerance [10], [14]. Class II AroA, which shares less than 30% amino acid identity with Class I AroA, has natural tolerance to glyphosate with high affinity for PEP [8], [15]. Currently, most commercial crop plants with glyphosate tolerance contain a Class II AroA from *Agrobacterium spp.* CP4. Several agronomic crops including maize and canola are transformed with both CP4 and glyphosate oxidase (GOX) genes together [16], [17].

From an extremely glyphosate-polluted environment in China, we have previously isolated a *Pseudomonas putida* strain (4G-1). A genomic library was generated, and subsequently screened in a glyphosate-sensitive *Escherichia coli* strain. As a result, a novel *aroA* gene (*PparoA*1) encoding a glyphosate-tolerant AroA was obtained [18], [19]. Phylogenetic analysis revealed that this AroA (PpAroA1) belongs to neither class I nor class II AroA enzymes. When compared with AroA from *Escherichia coli*, PpAroA1 from *Pseudomonas putida* showed a 300-fold-higher *K_i_*[glyphosate] value and similar *K_m_*[S3P] value and *K_m_*[PEP] value [18]. These favorable kinetic properties of this enzyme underpin a good potential of application in glyphosate-tolerant transgenic plant. In this study, attempts have been made to investigate if the optimized *aroA* gene (*PparoA*1_optimized_) and its coding enzyme (PpAroA1) from *Pseudomonas putida* can provide high tolerance to glyphosate in transgenic plants. Our results show that this novel PpAroA1 can efficiently confer tobacco plants with high tolerance to glyphosate.

## Results

### Generation of tobacco primary transformants

**Figure 1:**
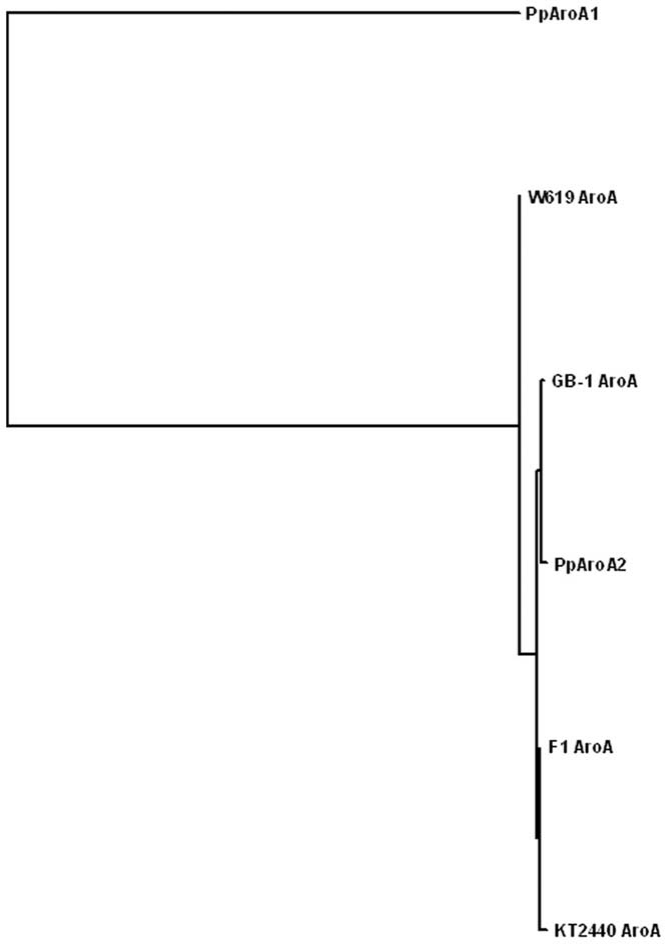
Phylogenetic analysis of *Pseudomonas* AroA proteins. PpAroA1 (AJ812018) and PpAroA2 (HM992507) are from *Pseudomonas putida* 4G-1. Other AroAs are from *Pseudomonas putida* F1 (NC_009512), KT2440 (NC_002947), GB-1 (NC_010322) and W619 (NC_010501). The phylogenetic tree was constructed using Maximum-Parsimony method in MAGE.

In previous report, we have identified a novel AroA from *Pseudomonas putida* 4G-1 with high tolerance to glyphosate, which showed little homology to class I as well as class II AroAs. Surprisingly, when compared with AroAs identified from other *Pseudomonas putida* strains, this novel AroA (designated as PpAroA1) showed very little (28%) identity to them [18]. Phylogenetic analysis indicated that PpAroA1 is evolutionary distant from other known *Pseudomonas* AroAs (Fig. 1).

In order to predict the expression of *PparoA*1 as a heterogenous gene in different host (for instance, tobacco in this study), codon adaptation index (CAI) was used in calculation [20]. The result showed that *PparoA*1 has a CAI value (∼0.7) in tobacco (*Nicotiana tabacum*), suggesting that it could be applied directly for generating transgenic plants in it (for details, see discussion). Moreover, the codon bias of the *PparoA*1 gene in tobacco was further adjusted by synthesizing a *PparoA*1_optimized_ gene. As a result, the *PparoA*1_optimized_ has got a more extreme codon bias, with a CAI value higher than the *PparoA*1 in tobacco (>0.9, Fig. S1).

**Figure 2:**
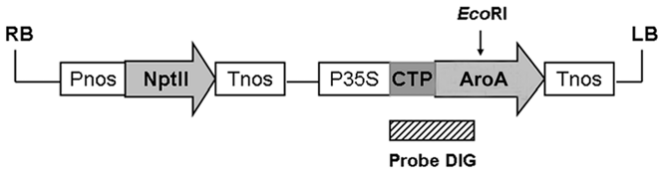
Structure of plasmid pBI121-*PparoA*1_optimized_. The gene cassette was cloned into pBI121 binary vector between the T-DNA left and right borders (LB and RB). Pnos, nopaline synthase promoter; P35S, cauliflower mosaic virus 35S promoter; Tnos, nopaline synthase terminator; NptII, neomycin phosphotransferase II gene; CTP, encoding sequence of chloroplast transit peptide from *Arabidopsis thaliana* AroA; AroA, *PparoA*1_optimized_ gene (synthetic sequence with codon usage adapted for dicots). Southern blot was hybridized with digoxigenin-labeled probe. Regions of homology are shown by rectangle in the schematic illustration.

In plant, AroA is a chloroplast-localized enzyme and the localization is directed by the amino terminal chloroplast transit peptide [21]. Therefore, the chloroplast transit peptide encoding sequence from *Arabidopsis thaliana* AroA was fused in frame with *PparoA*1_optimized_ gene. Subsequently, the above gene fusion was cloned into the binary T-DNA vector pBI121 [22]. As a result, the expression of the gene fusion was under the control of CaMV 35S promoter (the construct designated as pBI121-*PparoA*1_optimized_, Fig. 2). The construct was transferred into *tobacco* (*Nicotiana tabacum* L. cv. *W38*) leaf callus tissue via *Agrobacterium*-mediated transformation. To avoid false positive transformants that may be generated from direct screening for glyphosate tolerance, T-DNA mediated transformants were first screened for kanamycin resistance, a marker carried from the vector pBI121. In this case, 60 primary transformants containing *PparoA*1_optimized_ were obtained. Transgenic tobacco transformed with the empty vector pBI121 was used as negative control (designated as “vector control”) in this study.

### Obtaining transgenic tobacco plants with glyphosate tolerance

**Figure 3:**
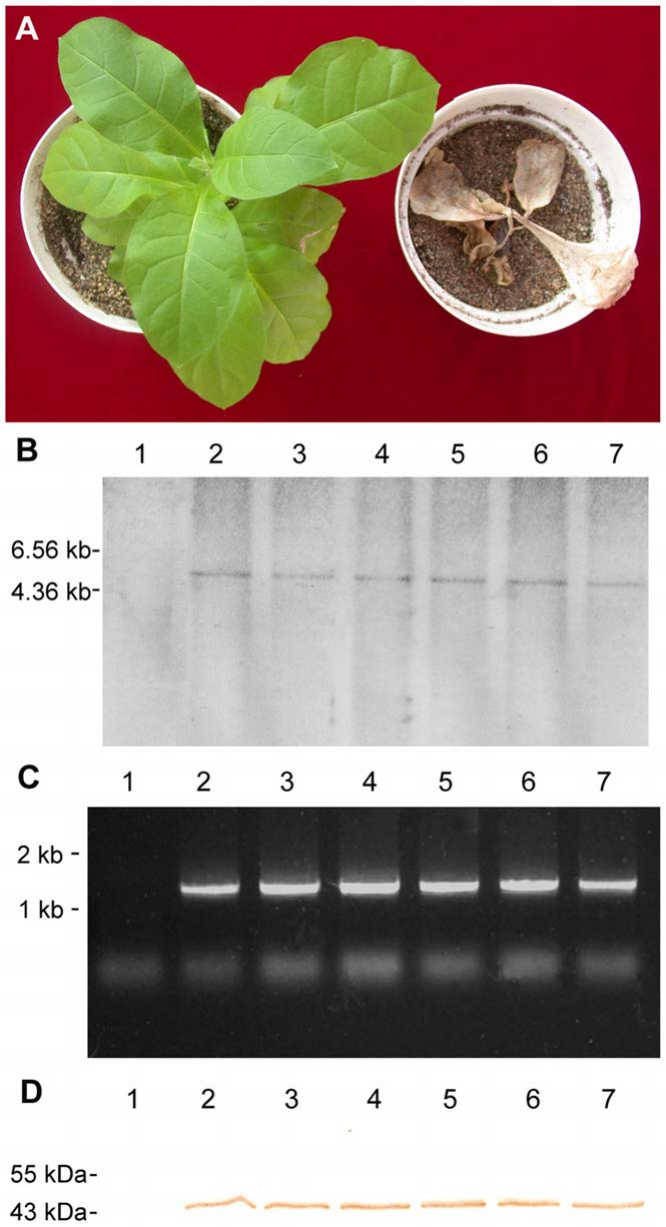
Analysis of transgenic tobacco with glyphosate tolerance. (A) Novel AroA confers glyphosate tolerance in tobacco. Plants were grown in a greenhouse under a condition with 16 h of light at 25°C and 8 h of darkness at 16°C. Glyphosate-tolerant plants (left) displayed normal healthy development. In contrast, “vector control” plant (right) did not survive. Plants are shown four weeks after spraying with Roundup™ glyphosate at a commercially recommended concentration. (B) Southern blot analysis of the *PparoA*1_optimized_ gene. A 50 µg aliquot of genomic DNA was digested with *Eco*RI, fractionated on an agarose gel, transferred to a nylon memebrane and probed with digoxigenin-labeled probe of *PparoA*1_optimized_ gene. (C) RT-PCR analysis on *PparoA*1_optimized_ gene expression in transgenic tobacco plants. (D) Western blot analysis of transgenic tobacco plants carrying *PparoA*1_optimized_ gene. Total protein extracted from leaves of transgenic lines was subjected to SDS-PAGE analyses. Molecular markers indicated the target protein to be the expected 47 kDa size. 1, control tobacco; 2–7, Transgenic Line Nt304, Nt305, Nt315, Nt316, Nt320, Nt323.

To measure the glyphosate tolerance, the above 60 primary transgenic tobacco lines containing *PparoA*1_optimized_ were tested by spraying Roundup™ with a commercial recommended dose. The results showed that six out of 60 primary transgenic tobacco lines survived with no leaf damage, one week after the treatment (Fig. 3A). These six transgenic tobacco plants were chosen for further studies (designated as Transgenic Line Nt304, Nt305, Nt315, Nt316, Nt320 and Nt323 respectively).

The following experiments were carried out on these transgenic lines. First, the presence of *AtCTP-PparoA*1_optimized_ gene fusion in these transgenic lines was confirmed by PCR (data not shown). Secondly, the copy number of T-DNA integration in these transgenic lines was determined by Southern blotting (Fig. 3B). In this case, only one hybridization band has been detected in glyphosate tolerant tobacco. In contrast, no hybridization could be detected in the “vector control” (Fig. 3B). Thirdly, RT-PCR was carried out, when *AtCTP-PparoA*1_optimized_ fusion-specific primers were used. The result showed a 1.5 kb fragment, corresponding to the right size of *AtCTP-PparoA*1_optimized_ fusion-transcript, which confirmed the inserted transgene and its transcription in the six glyphosate tolerant tobacco lines (Fig. 3C). As control, no amplification product could be detected in the empty vector transgenic plant. Finally, PpAroA1 rabbit polyclonal antibodies were generated (see material and methods), and used for western blotting of the PpAroA1 protein in total soluble cellular proteins isolated from the transgenic tobacco plants. The results showed a single band was observed in all six transgenic lines. The band indicated/demonstrated a molecular weight (approximately 47 kD) correspond to the mature PpAroA1 protein, without the chloroplast transit peptide. In contrast, no protein band was detected when leaf extracts from “vector control” tobacco (Fig. 3D). Taken together, the above results indicated that, in the six glyphosate tolerant transgenic lines, the *AtCTP- PparoA*1_optimized_ gene fusion was successfully transferred via *Agrobacterium*-mediated transformation. And its integration into the genome was as a single copy. This integrated gene fusion was actively transcribed with an expected RNA size (1.5 kb) to the *AtCTP-PparoA*1_optimized_ fusion-transcript. From this, RNA must have been translated and subsequently processed into a mature protein with a correspond size (47 kD) to the mature PpAroA1 protein.

### The glyphosate tolerant phenotype is stably inherited in T1 transgenic plants

**Figure 4:**
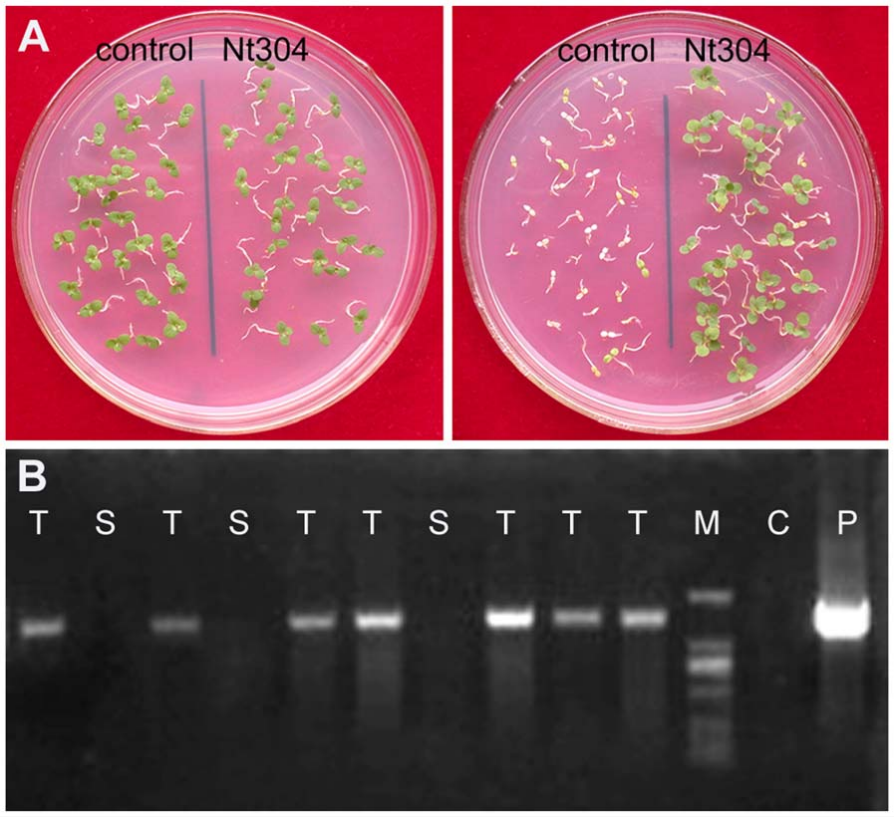
Analysis of T_1_ transgenic tobacco. (A) Seedlings germination assay. Seeds from “vector control” plant and transgenic line Nt304 were surface-sterilized and germinated on medium containing 0 (left) or 1 mM (right) glyphosate. The majority of Nt304 seedlings on the herbicide medium are green and healthy. However, all WT seedlings are bleached on glyphosate, indicating sensitivity. Photos were taken 3 weeks after plating seeds. (B) PCR analysis of T_1_ transgenic plants of Nt304. Progeny from T_0_ plants segregate into glyphoste-tolerant (T) and glyphosate-sensitive (S) individuals. M, DL2000 DNA marker (Tiangen, China); C, control tobacco; P, pBI121-*PparoA*1_optimized_.

In order to investigate the stability of the gene fusion and the phenotype in transgenic tobacco's progeny, T_1_ transgenic plants were then generated by crossing of the parental transgenic line. As the T_1_ seedlings of transgenic tobacco with the empty vector could not survive in the presence of 0.5 mM glyphosate (data not shown), the glyphosate tolerance of T_1_ transgenic seedlings were tested on MS medium containing 1 mM glyphosate. Statistic analysis indicated that about 75% transgenic tobacco seedlings of each line were found to be resistant to glyphosate. I. e., the ratio of segregation for seedlings tolerant to glyphosate versus sensitive to glyphosate was found to be close to 3∶1 (Table 1). This result indicated that segregation for glyphosate tolerance of self-crossed T_1_ transgenic tobacco seedlings was a typical Mendelian segregation. In contrast, the seedlings of “vector control” was inhibited and bleached by glyphosate treatment (Fig. 4A). Moreover, the T_1_ transgenic seedlings could still grow healthily when the concentration of glyphosate was increased to 10 mM. Therefore, the T_1_ transgenic tobacco seedlings showed at least 20 fold increase in glyphosate tolerance, when compared with “vector control” T_1_ seedlings.

**Table 1:** Segregation data for glyphosate tolerance of self-crossed T_1_ transgenic tobacco plants.

Line	Total	Tolerant	Sensitive	P	χ^2^
Nt304	296	216	80	0.727	0.122
Nt305	112	85	27	0.924	0.009
Nt315	246	183	63	0.924	0.009
Nt316	266	206	60	0.690	0.159
Nt320	100	75	25	1	0
Nt323	268	198	70	0.854	0.034

In order to investigate the correlation between phenotypic segregation and the segregation of *AtCTP-PparoA*1_optimized_ gene fusion among the T_1_ transgenic tobacco plants, Line Nt304 were selected for further analysis. Ten T_1_ transgenic tobacco plants of Line Nt304 were analyzed for the presence of *AtCTP-PparoA*1_optimized_ gene fusion in the genome DNA using PCR. A DNA band of 1521 bp, the identical size of to the chimeric gene, was amplified from genomic DNA of seven T_1_ transgenic plants, while no specific DNA band was observed from the other three T_1_ transgenic plants as well as the “vector control” plant (Fig. 4B). In parallel, these ten T_1_ transgenic plants were also treated with Roundup™ at a commercially recommended dose in a greenhouse. Results showed that the seven T_1_ transgenic plants with positive PCR detection all survived the treatment, while the three T_1_ transgenic plants with negative PCR detection were all sensitive to glyphosate. Therefore, it can be concluded that there is a direct correlation between glyphosate tolerance/sensitivity and the presence/absence of the transformed *AtCTP-PparoA*1_optimized_ gene fusion among the T_1_ transgenic tobacco plants respectively.

## Discussion

Previously, we have identified a novel AroA from *Pseudomonas putida* (designated as PpAroA1 in this study), naturally tolerant to high levels of glyphosate, has little homology with known AroAs [18]. PpAroA1, belongs neither to Class I nor to Class II AroAs. It is a novel AroA, and its sequence gets around patent protection [19].

In this study, we have shown that this PpAroA1 can confer transgenic tobacco plants with high tolerance to glyphosate. The evidence for this can be described in two ways. First, two steps screening of the transformants were carried out. 60 putative transformants were obtained on kanamycin containing plates, and 6 real transformants were obtained from these 60 putative transformants by spraying with glyphosate. This is further confirmed by molecular and biochemical characterization (Fig. 3). Secondly, a direct correlation between glyphosate tolerance/sensitivity and the presence/absence of the transformed *AtCTP-PparoA*1_optimized_ gene fusion among the T_1_ transgenic tobacco plants were obtained respectively. The segregation of glyphosate tolerance phenotype among the self-crossed T_1_ transgenic tobacco plants was a typical Mendelian segregation (Table 1). Therefore, we conclude that this PpAroA1 could be a good application candidate in transgenic crops with glyphosate tolerance in future.

**Figure 5:**
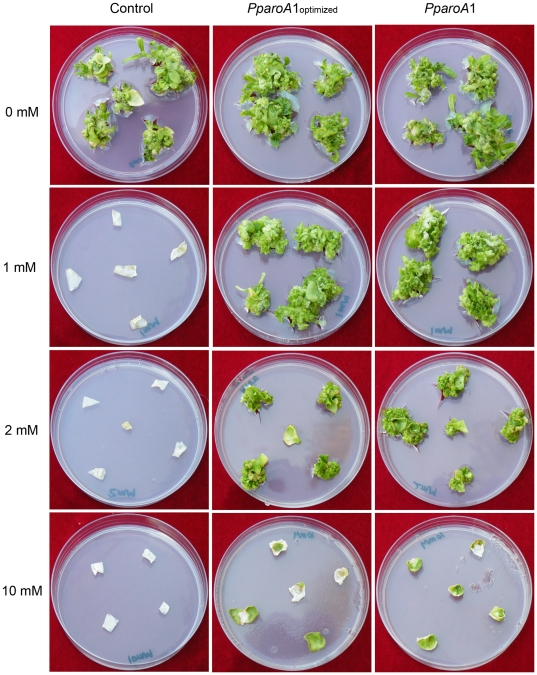
Glyphosate tolerance assay of transgenic tobacco with PpAroA1. *PparoA*1‐transgenic tobacco exhibited high tolerance to glyphosate, a level similar to that obtained from *PparoA*1_optimized_‐transgenic tobacco. Photograph was taken after one month of culture on medium containing 0–10 mM glyphosate. The left column shows leaf discs of “vector control” transgenic tobacco, the middle column shows leaf discs of *PparoA*1_optimized_ ‐transgenic tobacco, and the right column shows the leaf discs of *PparoA*1‐transgenic tobacco.

Besides the *PparoA*1 gene, the second gene encoding an alternative AroA (designed as PpAroA2) was cloned from *Pseudomonas putida* 4G-1 (for details, see material and methods). Two *aroA* genes were identified in *Pseudomonas putida* 4G-1 [18, this study]. The PpAroA1 showed very little (28%) identity to AroAs identified from other *Pseudomonas putida* strains [18]. In contrast, PpAroA2 contains 99% sequence identity to the AroA identified from *Pseudomonas putida* GB-1 (data not shown, for sequence see HM992507). Phylogenetic analysis indicated that PpAroA1 is evolutionary distant from other known *Pseudomonas* AroAs (Fig. 1). Therefore, the *PparoA*1 gene screened from *Pseudomonas putida* might come from a different species and inserted to the *Pseudomonas* bacteria through horizontal gene transfer. In addition, codon adaptation index (CAI) was used in calculation in order to predict the expression of *PparoA*1 as a heterogenous gene in different hosts [20]. The result showed that *PparoA*1 had higher CAI value, which indicated higher gene expression level in *Nicotiana tabacum* (∼0.7) than in *Pseudomonas putida* strain (∼0.3), suggesting that it could be applied directly for generating transgenic plants in tobacco (Fig. S1). Indeed, when the original *PparoA*1 gene from *Pseudomonas putida* was fused in frame with chloroplast transit peptide and introduced directly into the tobacco, it exhibited high tolerance to glyphosate, a level similar to that obtained from *PparoA*1_optimized_-transgenic tobacco (Fig. 5).

The transgenic tobacco plants with PpAroA1 exhibited high tolerance to glyphosate. Furthermore, the level of tolerance was comparable to that of agricultural application recommended by the manufacture [14], and similar to that achieved by *Agrobacterium spp.* CP4 AroA transgenic tobacco lines [8], [23]. In addition, the *PparoA*1 gene (as well as *PparoA*1_optimized_ gene) also has high CAI value in some crops such as maize, wheat and rice than the gene in *Pseudomonas putida*. This implies that the *PparoA*1 gene (as well as *PparoA*1_optimized_ gene) has got the potential to be highly expressed in these crops, and used to generate transgenic crop with glyphosate tolerance. Therefore, it indicates that the novel PpAroA1 is significant for generation of glyphosate-tolerant crop in the field of GM crops.

Future studies regarding the potential application of PpAroA1 will be undertaken in two ways. First, its function analysis in transgenic rice is underway. Secondly, since the function of PpAroA1 and its glyphosate tolerance could be reconstituted from its two protein fragments, divided in proper site [29, unpublished data], these characteristics may also be invaluable for the generation of transgenic crops resistant to glyphosate. Transgenic plants with a portion of the *aroA* gene in the chloroplast and the rest in targeted chromosomes would be a way to eliminate or reduce the possibility of the transgene migration.

## Materials and Methods

### Cloning the second aroA gene from Pseudomonas putida 4G-1

Genomic DNA from *Pseudomonas putida* 4G-1 was isolated and used as template to amplify another putative *aroA* gene (designated as *PparoA*2). Primers (*PparoA*2 F 5′-CCGGTGATGTGGCACGACAT-3′ and *PparoA*2 R 5′-TCACGACTT GGCCTCTTCTG-3′) were designed based on the conserved sequence of among all other *aroA* genes identified from *Pseudomonas putida* strains, including KT2440 (GI26986745), GB-1 (GI167031021), W619 (GI170719187) and F1 (GI148545259). The PCR product was sequenced. The resulting DNA sequence was submitted to Genbank (HM992507).

### Vector construction

For transformation in tobacco, the *Pseudomonas putida aroA* gene (*PparoA*1) was codon-optimized (designated as *PparoA*1_optimized_). The *PparoA*1_optimized_ gene was designed by us according to the codon bias of tobacco, and the gene was constructed using chemical synthetic methods (Shanghai Sangon, China), in which the codon usage was adapted to the codon bias of *Nicotiana tabacum*.

In order to target the protein to chloroplast, the chloroplast transit peptide from *Arabidopsis thaliana* AroA (AtCTP) was added at the N-terminus of PpAroA1. Two PpAroA1 encoding genes (*PparoA*1_optimized_ and *PparoA*1) were fused in frame with *AtCTP* respectively. Subsequently, the above gene fusions were cloned into *Bam*HI and *Sac*I sites of the binary T-DNA vector pBI121 [22], resulting in transformation construct pBI121-*PparoA*1_optimized_ and pBI121-*PparoA*1 respectively. The gene fusions replaced the *uid*A [encoding the β-glucuronidase (GUS)] gene of the original pBI121 vector, driven by the CaMV 35S promoter [22]. Transgenic tobacco transformed with the empty vector pBI121 was used as negative control (designated as “vector control”) in this study.

### Plant materials and plant transformation

Tobacco seeds (*Nicotiana tabacum* L. cv. *W38*) were kindly provided by Professor Zhong-Ping Lin (College of Life Science, Peking University, China). The seeds were sterilized with 15% NaClO for 15 min followed by five washes with sterile distilled water and germinated on solidified MS medium containing 3% sucrose, pH 5.8. The seedlings were used for transformation throughout this study.

Tobacco leaf sections were immersed in a bacterial culture of OD_600_∼0.3 for 15 min, dried on filter paper and transferred to MS [24] medium containing 1 mg/L 6-benzylaminopurine, 0.1 mg/L α-naphthalene acetic acid, 3% sucrose and solidified with 0.8% agar. After co-cultivation for 3 days at 25°C in darkness, the infected leaf sections were rinsed with water and transferred to regeneration medium (MS medium supplemented with 300 mg/L cefotaxime and 200 mg/L kanamycin). After regeneration, kanamycin-resistant shoots were selected and transferred to shoot elongation and root induction media (MS medium containing 0.1 mg/L *a*-naphthalene acetic acid, 300 mg/L cefotaxime and 200 mg/L kanamycin) [14].

### Glyphosate tolerance spray test

The tobacco transformants were propagated in sterile culture and then planted in soil in the greenhouse. The tobacco plants were sprayed with the herbicide Roundup™ (active ingredient isopropylamine salt of glyphosate, 41.0%), at the 5–6 leaf stage about 2 weeks after transplanting. The spray test was performed as described [23].

### PCR and Southern blot analysis

Tobacco genomic DNA was extracted from young leaves of transgenic and “vector control” plants by using the cetyl-trimethyl ammonium bromide (CTAB) method [25]. Integration of the desired gene into the tobacco genome was confirmed by PCR of the encoding sequence of the chloroplast transit peptide and PpAroA1. PCR was performed using the primers (*PparoA*1_optimized_ F 5′-ATGGCGCAAGTTAGCAGAATC-3′ and *PparoA*1_optimized_ R_1_ 5′-TCATGAGAA GTTGAATTGATG-3′), resulting in 1521 bp amplified DNA product (*AtCTP*- *PparoA*1_optimized_). The PCR products were analyzed by agarose gel electrophoresis.

For Southern blot analysis, 50 µg of tobacco genomic DNA was digested with *Eco*RI restriction enzyme (New England Biolabs Inc.). The digested DNA was separated on 0.8% (w/v) agarose gels and then transferred onto Hybond-N^+^ membrane (Amersham, UK) and cross-linked to the membrane by UV. The DIG-labeled probe was prepared by PCR using primers designed to amplify a 625 bp fragment from the *AtCTP-PparoA*1_optimized_ gene fusion (*PparoA*1_optimized_ F 5′- ATGGCGCAAGTTAGCA GAATC-3′ and *PparoA*1_optimized_ R_2_ 5′- CAAATGTGGAAGAATTTCATC-3′) (Fig. 1). Hybridization and immunological detection were performed with the DIG DNA labeling and detection kit (Roche, Germany).

### RNA extraction and transcript analysis

Total RNA of each sample was extracted from leaves of transgenic tobacco plants and “vector control” plant by using RNAprep pure Plant Kit (Tiangen, China). A 2 µg aliquot of RNA per sample was used to synthesize the first-strand cDNA by using M-MLV Reverse Transcriptase (Promega, USA) with random primer.

For detection of the transcripts of the *PparoA*1_optimized_ in tobacco plant, RT-PCR was performed using the same primers set as for the *AtCTP*-*PparoA*1_optimized_ gene fusion insert detection (*PparoA*1_optimized_ F and *PparoA*1_optimized_ R_1_). A PCR reaction was performed with the same primers without the reverse transcriptase step to demonstrate the absence of the genomic DNA contamination in the samples.

### Western blot analysis

Young leaf samples (100 mg) collected from transgenic and control tobacco plants were ground to powder in liquid nitrogen. The powders were suspended in 200 µl buffer B (0.25 M Tris HCl, pH 6.8, 8% 2-mercaptoethanol, 20% glycerol and 8% SDS). After lysis and centrifugation, the soluble fractions from leaf tissue were separated in 16% SDS polyacrylamide gel, and analyzed with immunoblots. The immunoblots were probed with 1∶2000 dilution of PpAroA1 rabbit polyclonal antibody. The antibody-antigen complex was visualized with alkaline phosphatase conjugated to goat anti-rabbit IgG (Promega, USA). In this study, the purified PpAroA1 was injected to the rabbit to produce the PpAroA1 rabbit polyclonal antibody in Institute of Genetics and Development Biology, Chinese Academy of Science.

### Seed germination assays

Transformation lines were grown in the greenhouse until maturity and seeds were harvested. Seed assays for resistance to glyphosate was performed as described [26]. T_1_ transgenic seedlings that are resistant to the glyphosate are uniformly green on the media, whereas sensitive seedlings are bleached.

## Supporting Information

Figure S1 **Codon adaptation index (CAI) values for** ***aroA*** **genes using different codon usage tables.** Pseudomonas, *Pseudomonas putida* KT2440; wheat, *Triticum aestivum*; maize, *Zea mays*; rice, *Oryza sativa*; tobacco, *Nicotiana tabacum*; tomato, *Lycopersicon esculentum*; pea, *Pisum sativum*; arabidopsis, *Arabidopsis thaliana*. Codon Adaptation Index (CAI) developed by Sharp and Li [20], is a measure of the synonymous codon usage bias for a DNA or RNA sequence and quantifies codon usage similarities between a gene and a reference set. The index ranges from 0 to 1, being 1 if a gene always uses the most frequently used synonymous codons in the reference set. CAI can be used for estimation of gene expressivity and giving an approximate indication of the likely success of heterologous gene expression [27]. CAI were calculated using EMBOSS and accordingly codon usage tables in the software suite [28].
(TIF)
