# Supplementary figures and images for: Novel AroA from Pseudomonas putida Confers Tobacco Plant with High Tolerance to Glyphosate
Source: PLoS One. 2011 May 18;6(5):e19732. doi: 10.1371/journal.pone.0019732 (PMC3097199; doi:10.1371/journal.pone.0019732)

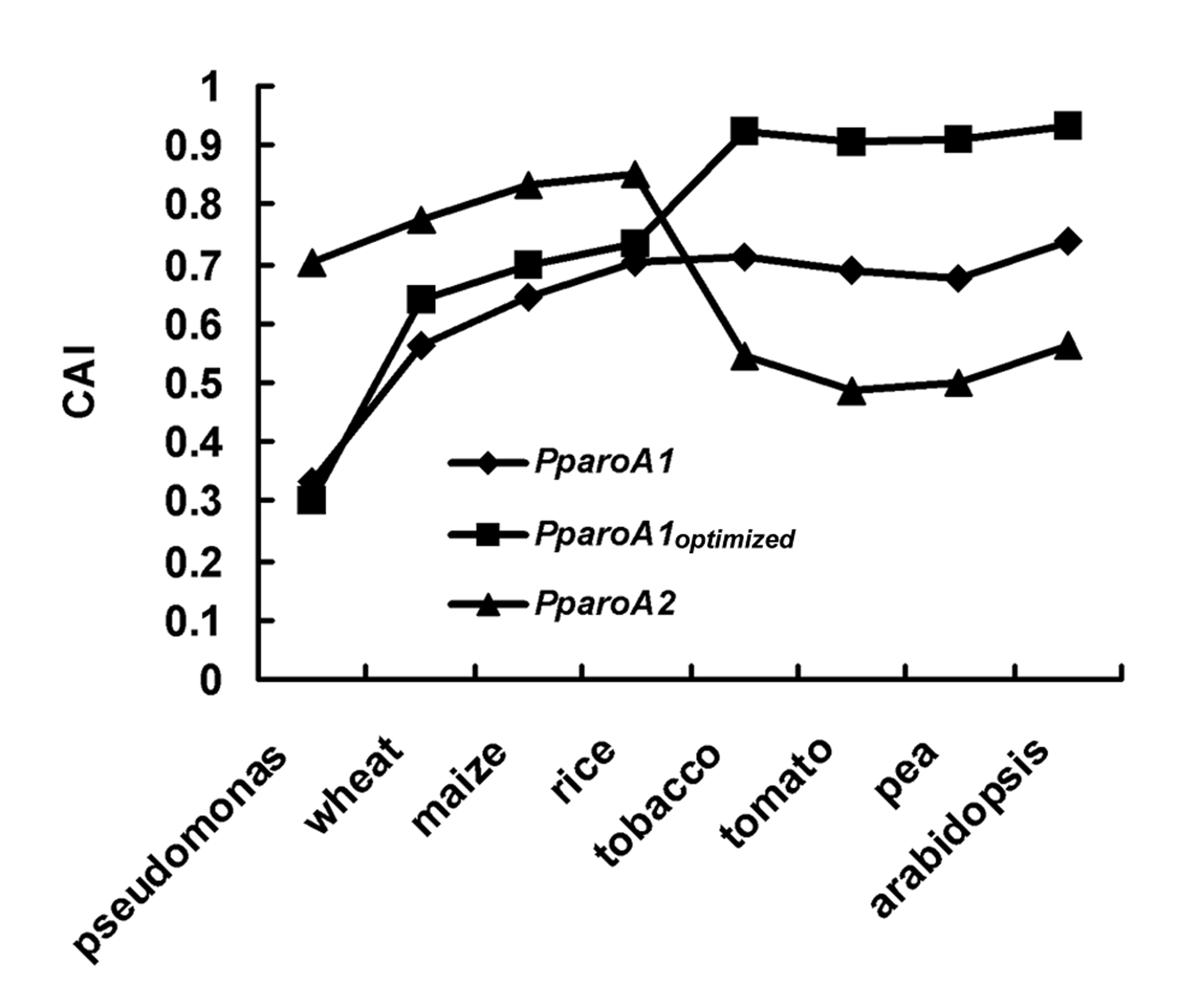

Supplement: Figure S1 — Codon adaptation index (CAI) values for aroA genes using different codon usage tables. Pseudomonas, Pseudomonas putida KT2440; wheat, Triticum aestivum; maize, Zea mays; rice, Oryza sativa; tobacco, Nicotiana tabacum; tomato, Lycopersicon esculentum; pea, Pisum sativum; arabidopsis, Arabidopsis thaliana. Codon Adaptation Index (CAI) developed by Sharp and Li [20], is a measure of the synonymous codon usage bias for a DNA or RNA sequence and quantifies codon usage similarities between a gene and a reference set. The index ranges from 0 to 1, being 1 if a gene always uses the most frequently used synonymous codons in the reference set. CAI can be used for estimation of gene expressivity and giving an approximate indication of the likely success of heterologous gene expression [27]. CAI were calculated using EMBOSS and accordingly codon usage tables in the software suite [28]. (TIF) [file pone.0019732.s001.tif]
